# Shared Fate of Meningeal Mast Cells and Sensory Neurons in Migraine

**DOI:** 10.3389/fncel.2019.00136

**Published:** 2019-04-05

**Authors:** Duygu Koyuncu Irmak, Erkan Kilinc, Fatma Tore

**Affiliations:** ^1^Department of Histology and Embryology, School of Medicine, Biruni University, Istanbul, Turkey; ^2^Department of Physiology, School of Medicine, Bolu Abant İzzet Baysal University, Bolu, Turkey; ^3^Department of Physiology, School of Medicine, Biruni University, Istanbul, Turkey

**Keywords:** migraine, neuroinflammation, mast cells, sensory neurons, ATP, CGRP, PACAP, autonomic nervous system

## Abstract

Migraine is a primary headache disorder which has complex neurogenic pathophysiological mechanisms still requiring full elucidation. The sensory nerves and meningeal mast cell couplings in the migraine target tissue are very effective interfaces between the central nervous system and the immune system. These couplings fall into three categories: intimacy, cross-talk and a shared fate. Acting as the immediate call-center of the neuroimmune system, mast cells play fundamental roles in migraine pathophysiology. Considerable evidence shows that neuroinflammation in the meninges is the key element resulting in the sensitization of trigeminal nociceptors. The successive events such as neuropeptide release, vasodilation, plasma protein extravasation, and mast cell degranulation that form the basic characteristics of the inflammation are believed to occur in this persistent pain state. In this regard, mast cells and sensory neurons represent both the target and source of the neuropeptides that play autocrine, paracrine, and neuro-endocrine roles during this inflammatory process. This review intends to contribute to a better understanding of the meningeal mast cell and sensory neuron bi-directional interactions from molecular, cellular, functional points of view. Considering the fact that mast cells play a *sine qua non* role in expanding the opportunities for targeted new migraine therapies, it is of crucial importance to explore these multi-faceted interactions.

## Introduction

Migraine headache has been known for 6,000 years but has not been completely cured yet. Human descriptions of migraine headache date from the earliest recorded history of man, in Mesopotamia, the cradle of civilization in 4000 B.C. In the 5th century, Hippocrates, the Father of Medicine, described in detail a headache that would be called migraine with aura. The Turkish philosopher physician Ibn-i Sina (930–1,037) also known as Avicenna, in his book The Canon of Medical Sciences, asserted that food and sounds provoked pain and that the patient could not tolerate light and should be rest alone in the darkness. This is still valid today for migraineurs (Daniel, 2010).

Today, migraine is listed in the top 20 conditions by the World Health Organization (WHO); a debilitating neurological condition with a high prevalence approximately 6%–8% of men and 15%–25% of women in western countries (Pietrobon and Striessnig, 2003). The Headache Classification Committee working under the International Headache Society, complete the following diagnostic criteria for migraine: episodic headache lasting from 4 to 72 h, concomitant with two of the following, throbbing, unilateral pain, aggravation on movement, or pain of at least moderate severity, and at least one of the following, nausea or vomiting, or photophobia and phonophobia (Headache Classification Subcommittee of the International Headache Society [IHS], 2004). Migraine is a condition that significantly impairs patients’ quality of daily life. The Global Burden of Disease Study ranked migraine as the seventh most common disabling pathology among 289 diseases, referred to as the 7th disabler (Wöber-Bingöl, 2013; Malone et al., 2015). Migraine has a co-morbidity in a number diseases which is mostly associated with mast cells (Graziottin et al., 2014; Xu and Chen, 2015; Eller-Smith et al., 2018; Graif et al., 2018).

To understand the pathophysiology of migraine, several important steps have been taken in the last decades. Today, it is known that the aura is caused by the activation of a neurophysiological phenomenon called cortical spreading depression (CSD) and the trigeminovascular system (Boran and Bolay, 2013; Alstadhaug, 2014). The mechanisms for triggering and ending the attacks are still largely mysterious.

In this review article, we aimed to compile the information about the migraine pathophysiology and the use of this data in efforts that have been made to find the best therapeutical options. In this regard, we focused on neurogenic inflammation which is crucial in the pathophysiology of migraine, mast cells, sensory neuronal axis and bi-directional interactions of these from molecular, cellular, functional and clinical points of views.

## Mast Cells

A medical student Paul Ehrlich (1878), coined the name “mastzellen” stemming from the Greek word mastos (μασδόσ), meaning breast (Crivellato et al., 2003). Today, we know that they originate from pluripotential hematopoietic cells in bone marrow and they are deeply involved in the trophism of tissues (Tore and Tuncel, 2009). Mast cells (MCs) are tissue-resident granulocyte that originate from CD34+/CD117+ and circulate in the blood during their immature stage (Tore and Tuncel, 2011; Theoharides et al., 2012). Stem cell growth factor (SCF) and other cytokines (Interleukin 3, 4, 9) help the maturation of MCs in the tissue (Varatharaj et al., 2012). MCs lodge in all vascularized tissues (3,000–25,000 mast cells/mm^3^), such as intestines, respiratory tract and skin as well as in the dura mater (Galli and Tsai, 2008; Theoharides et al., 2012).

Their vicinity enables MCs to be an “immediate call center” for exposure to pathogens and allergens. When activated, MCs degranulate and release mediators. They participate in tissue repair and interact with other immune cells (Galli and Tsai, 2008; Theoharides et al., 2012; Graziottin et al., 2014).

MCs are commonly round or elongated in shape, with a diameter of approximately 10–20 μm. They do not represent a homogeneous population. The recruitment and differentiation of MCs is a complex process in which tissue-specific microenvironmental factors such as cell types and cytokines, neuropeptides available around them are highly involved. Such cytokines and neuropeptides work through many up-and-down regulating mechanisms and are crucial in the recruitment, differentiation, maturation and even apoptosis of mast cells. The effect of a cytokine or neuropeptide on undifferentiated or mature mast cells might be different (Tore and Tuncel, 2011; Aich et al., 2015; Gupta and Harvima, 2018). MCs have proven to have fundamental differences in size, staining, sensitivity to stimuli/drugs, species, and function. The findings on these enigmatic cells are difficult to interpret.

**Figure 1:**
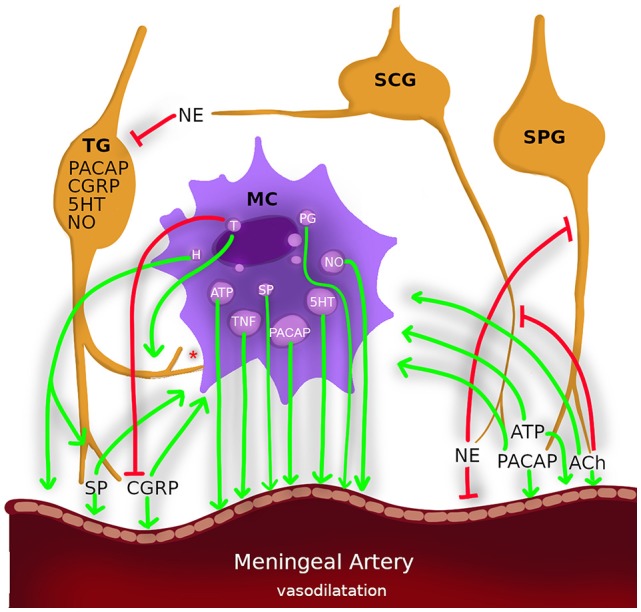
Nerves, mast cells and meningeal arteries constitute a triangle of paracrine/autocrine/endocrine interactions which involve the common fate in neurogenic inflammation in migraine. Nerves: trigeminal ganglion (TG), superior cervical ganglion (SCG), sphenopalatine ganglion (SPG). Neurotransmitters: calcitonin gene-related peptide (CGRP), pituitary adenylate cyclase activating polypeptide (PACAP), acetylcholine (ACh) and adenosine triphosphate (ATP) and substance P (SP) degranulate mast cells and causes vasodilatation (arrows). Mast cell mediators: histamine (H), nitric oxide (NO), and tumor necrosis factor-α (TNF-α) sensitizes sensory neurons and causes vasodilatation (arrows). Tryptase (T) also sensitizes sensory neurons (arrows) also cleavages CGRP (blinded lines). Norepinephrine (NE) inhibits trigeminal activation also causes vasoconstriction. *Mast cell have membrane-membrane contacts with sensory neurons.

Classical MC mediators are proteases (e.g., tryptase, chymase, etc.), bio-organic amines (e.g., histamine and serotonin), proteoglycans (e.g., heparin, etc.), many cytokines [e.g., tumor necrosis factor-α (TNF-α) nitric oxide (NO), prostaglandins, leukotrienes and kinins; Nilsson et al., 1999; Theoharides and Kalogeromitros, 2006; Gri et al., 2012; Sismanopoulos et al., 2012], neuropeptides [e.g., corticotropin-releasing factor, endorphins, somatostatin, substance P (SP), vasoactive intestinal peptide, Pituitary adenylate cyclase activating polypeptide (PACAP); Metcalfe et al., 1997; Luger and Lotti, 1998; Kempuraj et al., 2004; Theoharides and Kalogeromitros, 2006; Hildebrand et al., 2008; Lennerz et al., 2008; Tore and Tuncel, 2009; Okragly et al., 2018] as well as growth factors [e.g., transforming growth factor, vascular endothelial growth factor, granulocyte-monocyte colony stimulating factor, nerve growth factor (NGF); Metcalfe et al., 1997; Tore and Tuncel, 2009]. Each mast cell mediator function in a number of ways, and they overlap in their effects in the body. These mediators modify mast cell behaviors *via* autocrine effects, modulate neighboring nerves, endothelia and vessel smooth muscle functions *via* paracrine effects and regulate remote organ functions *via* endocrine effects (Figure 1).

Meningeal MCs and sensory nerve relation consist of proximity, communication and a shared fate. Proven not to be a random configuration, mast cell–nerve membrane-to-membrane contacts are highly common and in some cases, they even exchange granules (Rozniecki et al., 1999; Tore and Tuncel, 2011). This is likely to be by fate rather than an accident since such spatial distributions mostly indicate a functional relationship. MCs sometimes release their contents without degranulation; they also transport extracellular vesicles (30–150 nm) which contain several mediators, including micro RNAs and major histocompatibility complex-II, to the neighboring cells (Gupta and Harvima, 2018). MCs settle close to nerve fibers, which makes them strong candidates for modulating neural activity and nociception. Increased MC counts in proximity to the neural system (Barbara et al., 2004) and NGF are related with nerve fiber structure, which is responsible for hyperalgesia (Watson et al., 2008). MC degranulation also contributes to hyperalgesia in experimental settings (Vincent et al., 2013).

Sicuteri, in 1963 was one of the first to suggest a role for MCs in migraine pathophysiology by MC degranulating agent, compound 48/80 induced headache (Sicuteri, 1963). Plasma histamine, 5-hydroxy tryptamine (5-HT) and urinary histamine and tryptase levels are elevated during migraine attacks (Heatley et al., 1982; Ferrari et al., 1989; Olness et al., 1999). Histamine infusion also promotes a migraine-like headache (Lassen et al., 1995). MCs are not the only source for histamine in brain; specifically 90% of thalamic histamine and up to 50% of total brain histamine are produced by MCs in rats (Dong et al., 2014). Histamine receptors (H1, H2, H3, and H4) are expressed in microglias, astrocytes, sensory neurons, smooth muscle cells of vessels, and also in MCs itself. These receptors mediate or prevent degranulation of MCs in certain conditions in related venues (Rosa and Fantozzi, 2013; Alstadhaug, 2014). Cromolyn and antihistaminics were used for migraine prophylaxis (Rossi et al., 2003). But only a small proportion of migraineurs have benefited from them, possibly due to the variety of mast cell responses and mast cell mediator contents. As illustrated in Figure 1, not only histamine but also many mast cell mediators are involved, directly or indirectly, in the activation of meningeal nociceptors (Levy et al., 2007; Zhang et al., 2007; Zhang and Levy, 2008; Rosa and Fantozzi, 2013). Recently Kilinc et al. (2017). showed that compound 48/80 induced persistent nociceptive firing in the trigeminal nerve endings was blocked by 5-HT3 receptor antagonist.

## Neurogenic Inflammation

In recent decades, emerging data from animal and human research brought the integrated theory which implicates vascular and neural components. In particular, the activation of the meningeal afferent neurons, neuropeptide release, and neurogenic inflammation play key and complex roles in migraine headache (Buzzi and Moskowitz, 2005; Peroutka, 2005; Burgos-Vega et al., 2015). This concept has been supported by the large amount of experimental evidence accumulated. The studies were mainly performed by the activation of primary afferent neurons, either in disease or disease mimicking condition or experimentally with electrical stimulus, or by the activation of polymodal nociceptive receptors expressed on the peripheral nerve terminal that causes the release of proinflammatory neuropeptides. In this framework, these neuropeptide mediators interact with endothelial cells, mast cells, immune cells, and vascular smooth muscle cells, initiating a cascade of inflammatory responses (Geppetti et al., 2012).

However, there is not a full consensus on the pathophysiology of migraine, though it has been agreed that this disorder can be said mainly to result from the activation and sensitization of the trigeminovascular system (Goadsby et al., 2017a,b). Activated trigeminal nociceptive afferents release calcitonin gene-related peptide (CGRP) and SP, which subsequently cause sterile neurogenic inflammation in the meninges. Neurogenic inflammation is characterized by vasodilatation of meningeal vessels, increased vascular permeability, plasma protein extravasation, and mast cell degranulation (Boran and Bolay, 2013; Erdener and Dalkara, 2014). Although CGRP or some other vasodilatators of meningeal arteries do not induce nociceptive activation, vasodilatation of extracranial vessels can activate nociceptive afferents (Levy et al., 2005; Shevel, 2011). It has been proposed that the inflammatory mediators further activate meningeal nociceptors and induce peripheral and central sensitization (Levy, 2012).

Under experimental conditions, neurogenic inflammation can be induced by inflammatory agents applied topically to the dura mater. An inflammatory cocktail containing histamine, serotonin, bradykinin, and prostaglandin E2, was used to elucidate migraine pathophysiology and to predict the effectiveness of the treatments that have been developed for migraine (Zhang and Levy, 2008; Yan et al., 2018). A functional magnetic resonance imaging study showed that dural application of the inflammatory cocktail in awake rats demonstrated similar responses to migraine patients (Becerra et al., 2017). Meningeal inflammation arises as a result of CSD. During CSD, mediators, such as potassium-ions and glutamate are released and can cause the activation of nociceptors on meningeal sensory neurons and mast cells (Waeber and Moskowitz, 2005; Bogdanov et al., 2011; Karatas et al., 2013; Pietrobon and Moskowitz, 2013). This framework suggests a cross-talk of the actors such as MCs and neurons in meningeal inflammation. Ramachandran has recently outlined the mechanistic hypothesis of neurogenic inflammation in the dura mater (Ramachandran et al., 2014). He proposed that CSD or stress factors lead events along two separate paths. Either the trigeminal system is activated and neuropeptides are released, or the MCs are degranulated and sensitize the nociceptors. In both cases, neuropeptides such as CGRP and many MC mediators induce meningeal vasodilatation (Figure 1). In neurogenic inflammation, where MCs, sensory nerves and blood vessels form a multifaceted triangle (Figure 1). MC releases algogenic and vasoactive mediators, which activate sensory nerve fibers and cause vasodilatation *via* paracrine, autocrine and neuroendocrine interactions (Aich et al., 2015; Tore and Tuncel, 2011; Theoharides et al., 2012; Gupta and Harvima, 2018). Then, nerve fibers release inflammatory and vasoactive neuropeptides; they activate mast cells which results in a vicious cycle of mast cell and nociceptor activation leading to neurogenic inflammation and pain. Like a chicken and egg situation, we still do not know which comes first: nociceptor activation or mast cell activation. Is there a key molecule that activates both of them at the same time? This situation plays an important role not only in the pathogenesis of migraine but also of numerous mast cell-associated diseases including asthma, fibromyalgia, eczema, psoriasis, interstitial cystitis, liver fibrosis, inflammatory bowel diseases, colitis, periodontitis, and arthritis (Tore and Tuncel, 2011; Theoharides et al., 2012). Surprisingly, the literature on the etiopathogenesis of these diseases discuss the same cells, the same receptors, and the same clinical management recommendations; however, there is a gap in the correlation of all this data concerning specific aspects of MCs involvement.

## From Functional Cross-Talk Toward Therapeutical Target

### Autonomic Nervous System, the Oldest

MCs are found mostly in cranial dura mater which is innervated densely by both autonomic and sensory nerves. Autonomic parasympathetic nerves originate from the sphenopalatine and otic ganglia, whereas sympathetic nerves originate from the superior cervical ganglion (SCG). These neurons express PACAP, NO, vasoactive intestinal polypeptide (VIP), norepinephrine (NE), acetylcholine (ACh), and neuropeptide Y (NPY; Artico and Cavallotti, 2001; Goadsby, 2013; Levy et al., 2018). It has been long suspected that the autonomic nervous system might play a role in the pathophysiology of migraine (Peroutka, 2004; Alstadhaug, 2009; Goadsby, 2013). An autonomic dysfunction has been repeatedly described in headache sufferers. ACh was the first discovered neurotransmitter and the first postulated neurotransmitter in migraine pathophysiology because of its vasodilatory and pain-inducing effects. Almost a hundred years ago, for the first time, Kunkle showed increased ACh levels in the cerebrospinal fluid (CSF) of migraine patients (Kunkle, 1959). Later, this approach was displaced from the attention of the researchers by the rise of the serotonin theory. Recently, some studies have recalled attention to the parasympathetic system in migraine. Parasympathetic contributions to the peripheral and central sensitization during migraine have been reported (Yarnitsky et al., 2003). CSD induces trigeminal and parasympathetic activation (Bolay et al., 2002). Giniatullin group showed that ACh, carbachol and nicotine significantly increased nociceptive firing in the peripheral terminals of the meningeal trigeminal nerves (Schytz, 2010; Mikhailov et al., 2016; Shelukhina et al., 2017). Carbachol, but not nicotine, induced massive degranulation of meningeal mast cells. In a clinical study report, sphenopalatine ganglion blockage prevented migraine attacks (Binfalah et al., 2018).

The sympathetic nervous system inhibits the trigeminal system (Peroutka, 2004). Superior cervical ganglionectomy increased dura mater NO levels, c-fos expression in the spinal trigeminal nucleus caudalis (TNc) and induced degranulation of meningeal mast cells (Tore et al., 2010; Kilinc et al., 2015). Yildiz et al. (2007) measured a facial sympathetic skin response that indicated the activation of the sympathetic nervous system. They found a sympathetic hypofunction on the symptomatic side in attack and interictal periods and contrary in the post-attack period in migraineurs (Yildiz et al., 2008). Sympathetic hypofunction might lead to an increase in parasympathetic activation, or vice versa, because of reciprocal innervations of the autonomic nervous system in many organs. Recently, the meta-analysis showed a major catecholamine metabolite homovanillic acid (HVA) increase in CSF obtained from migraineurs compared to controls (van Dongen et al., 2017). While the roles of serotonin and CGRP in migraine treatment are popularly considered, the involvement of the autonomic nervous system has been ignored in clinical trials. However, we suggest that it should be kept in mind as a new therapeutic approach.

### CGRP, the Most Popular

CGRP is a 37-amino acid neuropeptide neurotransmitter that was first identified in 1982 (Amara et al., 1982). CGRP and its receptors are found in all organs, especially sensory neurons. CGRP is highly expressed in the central terminals of the trigeminal nerve and the trigeminal ganglion (TG) where CGRP is often coreleased with SP. CGRP is multifunctional peptide mediating pain as well as a growth factor for primitive cells, Schwann cells and endothelial cells (Lennerz et al., 2008; Recober and Russo, 2009; Messlinger, 2018).

Previously, the effects of CGRP on the cardiovascular system were studied intensively. CGRP is known to regulate cardiac excitability, microvascular permeability, vascular smooth muscle tone, and angiogenesis. CGRP is a potent dilator of cerebral and dural vessels (Brain and Grant, 2004). A meta-analysis showed increased concentrations of CGRP in the CSF and blood of migraineurs. The plasma CGRP level is proposed as a biochemical biomarker for migraine (Cernuda-Morollón et al., 2013). Infusion of CGRP can trigger a migraine attack (van Dongen et al., 2017). Stimulation of human TG increases CGRP levels in cranial circulation (Goadsby et al., 1988). Additionally, CGRP can also cause degranulation and subsequent release of inflammatory mediators from meningeal mast cells, whereas MC mediator histamine does not induce CGRP release in meningeal sensory afferents (Theoharides et al., 2005; Russo and Dickerson, 2006; Schwenger et al., 2007; Lennerz et al., 2008). There is a complex bidirectional relationship between MCs and CGRP. Mast cell tryptase not only activates proteinase-activated receptor 2 (PAR2) in trigeminal nociceptive afferent nerves which results in the release of NO, SP and CGRP but also cleavages CGRP (Zhang and Levy, 2008; Dux et al., 2009; Tore et al., 2010). PAR2 and transient receptor potential vanilloid 1 (TRPV1) receptors are colocalized in dural afferents and the sensitization of TRPV1 receptors by PAR2 mediates CGRP release (Dux et al., 2009; Zakharov et al., 2015). NO sources can be neurons, mast cells, and endothelial cells. Wherever it comes from, NO triggers perivascular neurogenic inflammation by facilitating the synthesis and release of CGRP and SP from dural nociceptive afferent fibers (Olesen, 2008). NO promotes CGRP gene activity in trigeminal neurons, and it works by signaling *via* a mitogen-activated protein (MAP) kinase pathway and T-type calcium channels. This suggests that endogenous NO could have a modulatory role in neurogenic inflammation (Ramachandran et al., 2014).

Recently Kilinc et al. (2017) indicated that 5-HT induced CGRP release and nociceptive activity in peripheral nerve terminals *via* 5-HT3 receptors. 5-HT is pro-nociceptive peripherally and anti-nociceptive centrally (Kilinc et al., 2017). The same author showed that calcitonin administration prevented CGRP release, trigeminal activation and mast cell degranulation in a glyceryltrinitrate-induced migraine model and* ex vivo* meningeal preparations (Kilinc et al., 2018). CGRP cannot easily pass the blood-brain barrier. Thus, it may induce the generation of pronociceptive substances and receptors in the trigeminal ganglion, transported along the central terminals. In this way, peripherally acting therapeutics can have a central antinociceptive effect (Messlinger, 2018). The rationale constituting the basis for this target can be at least based on CGRP receptor locations, the activation mechanism of these receptors and the change in the levels of this peptide in relevant venues.

### PACAP, the Newest

PACAP, which was first isolated from ovine hypothalamic extracts, is a new player in the migraine arena. It was named for its action, which is to stimulate cAMP formation in anterior pituitary cells (Dogrukol-Ak et al., 2004; Eftekhari et al., 2015). Two amidated forms with PACAP38 and PACAP27 residues exist, but the major form in tissues is PACAP38, with high concentrations found in the trigeminal ganglion, hypothalamus, cerebral cortex, hippocampus, posterior pituitary, testes and adrenal gland (Eftekhari et al., 2015). PACAP38 (10 pmol/kg/min) induces migraine-like attacks in patients with migraine without aura (Schytz, 2010). In migraineurs, the level of PACAP in the peripheral blood is increased during a migraine attack (Tuka et al., 2013). PACAP induces marked vasodilation and degranulation of dural mast cells (Baun et al., 2012). MCs release PACAP itself (Okragly et al., 2018). Körtési et al. showed that electrical stimulation of the TG increased mRNA expression of PACAP38 which was inhibited by N-methyl-d-aspartate (NMDA) glutamate (NMDA) receptor inhibitor, kynurenic acid or MK-801 (Tuka et al., 2012; Körtési et al., 2018). PACAP is transported into the brain by transmembrane diffusion, a non-saturable mechanism (Dogrukol-Ak et al., 2004). Not only neuronal PACAP but also mast cell-derived PACAP can be involved in migraine pathophysiology.

### ATP, the Last but Not the Least

The nucleotide adenosine triphosphate (ATP) is a promising candidate for migraine pathophysiology. ATP is well known as an intracellular energy source, however accumulating data proved that ATP is also a neurotransmitter (Burnstock, 1981, 2006; Burnstock et al., 2011). Extracellular ATP exhibits its effects *via* the two main types of purinergic receptors: ionotropic P2X (P2X1-7) and metabotropic P2Y (P2Y1-14). Rat mast cells express both ATP and cell-surface purinergic receptors of the P2 class; therefore, they are both the source and target of extracellular ATP (Bulanova and Bulfone-Paus, 2010; Burnstock and Boeynaems, 2014; Idzko et al., 2014). It was demonstrated that the activation of P2X7 receptors with both ATP and BzATP (P2X/agonist) increases calcium in human mast cells and induces degranulation of mast cells (Bulanova and Bulfone-Paus, 2010; Arandjelovic et al., 2012; Wareham and Seward, 2016). It was also shown that extracellular ATP evoked nociceptive spikes through P2X3 receptors in trigeminal nerve fibers innervating meninges at the origin site of migraine pain (Yegutkin et al., 2016). Additionally, CGRP upregulates P2X3 receptors in trigeminal sensory neurons (Fabbretti et al., 2006). Recently, Giniatullin’s group has demonstrated that extracellular ATP promotes the activation of trigeminal neurons and degranulation of meningeal mast cells *via* P2X7 receptors (Nurkhametova et al., 2019). ATP might be a key molecule responsible for the vicious cycle between meningeal mast cells and the nervous system. The antagonists of P2X3 and P2X7 receptors maybe promising potential targets for migraine treatment.

## Clinical Trials and Recent Approaches

Migraine undoubtedly has a severely disabling nature, thus there is an inevitable unmet medical need here as we still do not have a fully effective and safe treatment. Existing therapies are often non-specific, poorly tolerated, not fully effective or have cardiovascular contraindications, resulting in limitations on the use of these treatments. Among the issues seen, in turn, at patients’ level, it is notable that half of the patients are not satisfied with current therapies in terms of pain recurrence, the same percentage complainants of requiring supplementary dosing, almost 80% of the patients consider acute alternative immediate therapies, and medication overuse headache also accompanies the therapies (Tepper, 2018).

In the 1990s, serotonin 5-HT1B/1D receptor agonists were introduced for improvement in the management of acute migraine, and there have been new trials investigating new agonists that are effective and result in fewer or no adverse events. LY-334370, a selective serotonin-1F-receptor agonist, has been reported to be efficacious in the abortive treatment of migraine (Shepheard et al., 1999) as it acts by inhibiting neurogenic inflammation. Moreover, new compounds like 4991W93 (Earl et al., 1999) and PNU-14263 (Gomez-Mantilla et al., 2001) are selective serotonin-1D-receptor agonists.

As a promising agent, NGF-targeted therapies using NGF-sequestering antibodies were highly effective in pain control, but as these compounds led the adverse events involving the sympathetic nervous system and bones, this treatment option was stopped by the Food and Drug Administration (FDA; Kelleher et al., 2017; Skaper, 2017; Gupta and Harvima, 2018).

Some trials have demonstrated the nonselective NO synthase inhibitor, L-N-monomethylarginine (L-NMMA), to be highly successful in treating both migraine attacks and chronic tension-type headache (Ashina, 2002). NO synthase inhibitors may open the way to new avenues in the pharmacological treatment of migraine as they act by inhibiting NO production and neuropeptide release and pharmacological inhibition of several steps of the NO-signaling cascade. Cotreatment with the serotonergic, antimigraine drug sumatriptan suppresses the stimulatory effects of NO on CGRP promoter activity and release. Similarly, the application of nonselective and neuronal nitric oxide synthase (nNOS) inhibitors was able to partially attenuate neurogenic vasodilation (Klede et al., 2003).

Acting as a pivotal player in migraine pathophysiology, CGRP has been defined as a therapeutic target for migraine therapy. This attraction towards CGRP comes from its role both in onset and probably in the progress of the disease (Tepper, 2018). The translation of the CGRP acting mechanism at the beginning and during the course of the migraine into a therapeutical approach led to two paths: the development of small molecule CGRP receptor antagonists (Gepants), and the development of monoclonal antibodies. The gepants which have been the subject of published trials for acute treatment of migraine are as follows: Olcegepant (Olesen et al., 2004), Telcagepant (Ho et al., 2008; Connor et al., 2009), Rimegepant (Marcus et al., 2014), Ubrogepant (Voss et al., 2016), BI 44370 TA (Diener et al., 2011), MK-3207 (Hewitt et al., 2011). A severe adverse event liver toxicity appeared as a major challenge in the development programs of the gepants which was well-documented in the clinical trials, mostly tested through phase II, and phase III trials. The said drug development program was terminated due to the liver toxicity signals (Connor et al., 2011). Gepants are still important in identifying CGRP as key in migraine treatment and a potential target for acute treatment and, maybe for prophylaxis. It is well-understood that gepants are effective in the acute treatment of episodic migraine. The evidence also reveals that they are well tolerated. Ongoing and future studies on gepant safety, tolerability, and efficacy in migraine prevention are currently being evaluated. The data obtained from phase II and III trials of eptinezumab, erenumab, fremanezumab, and galcanezumab demonstrated that these monoclonal antibodies targeting the CGRP pathway demonstrate favorable effects in the preventive treatment of episodic and chronic migraine. A number of phase II and III trials are being conducted to further determine or prove the efficacy and safety of this new drug option. The cardiovascular effects of long-term CGRP blockade should be taken as a priority (Khan et al., 2019; www.ClinicalTrials.gov).

In particular, the most popular large phase III studies demonstrated that treatment with erenumab was associated with a substantive decrease in migraine frequency and the requirement for acute migraine-specific medication use in patients with episodic migraine. These trials are well reported as follows: ARISE (NCT02483585; Dodick et al., 2018), STRIVE (NCT02456740; Goadsby et al., 2017a,b) and LIBERTY (NCT03096834; Reuter et al., 2018). STRIVE and ARISE were completed within the first half of 2017. Positive results for erenumab constituted the basis for its recent approval in the US for the preventive treatment of migraine in adults. It has also received a favorable opinion in the Europen Union (EU) for the prophylaxis of migraines.

There are a few trials investigating the corticosteroid treatments that can be associated with MC involvement in this context, i.e., NCT02903680, NCT03220113, NCT03066544 (www.ClinicalTrials.gov).

## Conclusion

Mast cell-sensory nerve relationship in migraine pathophysiology is versatile and not fully mapped, yet. The main difficulty in understanding these complex interactions that constitute the bi-directional cross-talk of the mast cells, nerves and the vessel components comes from multi-dimensional channels of communication working in harmony or disharmony. The diverse interpretation of the messages that are released from dose-dependent ligand response constitutes a wide spectrum of commentary remarks. Thus, the clinical evidence suggests that there is no one-fit-all treatment choice for migraine.

We suggest hereby, to further understand the etiopathogenesis and molecular aspects of migraine, specifically neurogenic inflammation. More detailed identification of the disease sub-types, plus a better understanding of the individual conditions will help scientists to find and the physicians to decide on the treatment choices tailored for the patients. In order to investigate the effective and safe treatment options which entail the combination of the existing monoclonal antibodies and mast cell stabilizers and triptans for the proper combat with the least side effects and the most efficacious cure methods could be evaluated.

## Author Contributions

All authors have equal role in literature search, writing and revising the manuscript.

## Conflict of Interest Statement

The authors declare that the research was conducted in the absence of any commercial or financial relationships that could be construed as a potential conflict of interest.

## References

[B1] Aich A. Afrin L. B. Gupta K. (2015). Mast cell-mediated mechanisms of nociception. Int. J. Mol. Sci. 16, 29069–29092. 10.3390/ijms161226151 26690128 PMC4691098

[B2] Alstadhaug K. B. (2009). Migraine and the hypothalamus. Cephalalgia 29, 809–817. 10.1111/j.1468-2982.2008.01814.x 19604254

[B3] Alstadhaug K. B. (2014). Histamine in migraine and brain. Headache 54, 246–259. 10.1111/head.12293 24433203

[B4] Amara S. G. Jonas V. Rosenfeld M. G. Ong E. S. Evans R. M. (1982). Alternative RNA processing in calcitonin gene expression generates mRNAs encoding different polypeptide products. Nature 298, 240–244. 10.1038/298240a0 6283379

[B5] Arandjelovic S. McKenney K. R. Leming S. S. Mowen K. A. (2012). ATP induces protein arginine deiminase 2-dependent citrullination in mast cells through the P2X7 purinergic receptor. J. Immunol. 189, 4112–4122. 10.4049/jimmunol.1201098 22984079 PMC3466374

[B6] Artico M. Cavallotti C. (2001). Catecholaminergic and acetylcholine esterase containing nerves of cranial and spinal dura mater in humans and rodents. Microsc. Res. Tech. 53, 212–220. 10.1002/jemt.1085 11301496

[B7] Ashina M. (2002). Nitric oxide synthase inhibitors for the treatment of chronic tension-type headache. Expert Opin. Pharmacother. 3, 395–399. 10.1517/14656566.3.4.395 11934342

[B8] Barbara G. Stanghellini V. de Giorgio R. Cremon C. Cottrell G. S. Santini D.. (2004). Activated mast cells in proximity to colonic nerves correlate with abdominal pain in irritable bowel syndrome. Gastroenterology 126, 693–702. 10.1053/j.gastro.2003.11.055 14988823

[B9] Baun M. Pedersen M. H. Olesen J. Jansen-Olesen I. (2012). Dural mast cell degranulation is a putative mechanism for headache induced by PACAP-38. Cephalalgia 32, 337–345. 10.1177/0333102412439354 22421901

[B10] Becerra L. Bishop J. Barmettler G. Kainz V. Burstein R. Borsook D. (2017). Brain network alterations in the inflammatory soup animal model of migraine. Brain Res. 1660, 36–46. 10.1016/j.brainres.2017.02.001 28167076 PMC5731648

[B11] Binfalah M. Alghawi E. Shosha E. Alhilly A. Bakhiet M. (2018). Sphenopalatine ganglion block for the treatment of acute migraine headache. Pain Res. Treat. 2018:2516953. 10.1155/2018/2516953 29862074 PMC5971252

[B12] Bogdanov V. B. Multon S. Chauvel V. Bogdanova O. V. Prodanov D. Makarchuk M. Y.. (2011). Migraine preventive drugs differentially affect cortical spreading depression in rat. Neurobiol. Dis. 41, 430–435. 10.1016/j.nbd.2010.10.014 20977938

[B13] Bolay H. Reuter U. Dunn A. K. Huang Z. Boas D. A. Moskowitz M. A. (2002). Intrinsic brain activity triggers trigeminal meningeal afferents in a migraine model. Nat Med. 8, 136–142. 10.1038/nm0202-136 11821897

[B14] Boran H. E. Bolay H. (2013). Pathophysiology of migraine. Arch Neuropsychiatry 50, 1–7. 10.4274/Npa.y7251 PMC5353071 28360576

[B15] Brain S. D. Grant A. D. (2004). Vascular actions of calcitonin gene-related peptide and adrenomedullin. Physiol. Rev. 84, 903–934. 10.1152/physrev.00037.2003 15269340

[B16] Bulanova E. Bulfone-Paus S. (2010). P2 receptor-mediated signaling in mast cell biology. Purinergic Signal. 6, 3–17. 10.1007/s11302-009-9173-z 19921464 PMC2837823

[B17] Burgos-Vega C. Moy J. Dussor G. (2015). Meningeal afferent signaling and the pathophysiology of migraine. Prog. Mol. Biol. Transl. Sci. 131, 537–564. 10.1016/bs.pmbts.2015.01.001 25744685

[B18] Burnstock G. (1981). Pathophysiology of migraine: a new hypothesis. Lancet 1, 1397–1399. 10.1016/s0140-6736(81)92572-1 6113355

[B19] Burnstock G. (2006). Historical review: ATP as a neurotransmitter. Trends Pharmacol. Sci. 27, 166–176. 10.1016/j.tips.2006.01.005 16487603

[B20] Burnstock G. Boeynaems J. M. (2014). Purinergic signalling and immune cells. Purinergic Signal. 10, 529–564. 10.1007/s11302-014-9427-2 25352330 PMC4272370

[B21] Burnstock G. Fredholm B. B. Verkhratsky A. (2011). Adenosine and ATP receptors in the brain. Curr. Top. Med. Chem. 11, 973–1011. 10.2174/156802611795347627 21401499

[B22] Buzzi M. G. Moskowitz M. A. (2005). The pathophysiology of migraine: year 2005. J. Headache Pain 6, 105–111. 10.1007/s10194-005-0165-2 16355290 PMC3451639

[B23] Cernuda-Morollón E. Larrosa D. Ramón C. Vega J. Martínez-Camblor P. (2013). Pascual interictal increase of CGRP levels in peripheral blood as a biomarker for chronic migraine. Neurology 81, 1191–1196. 10.1212/wnl.0b013e3182a6cb72 23975872

[B24] Connor K. M. Aurora S. K. Loeys T. Ashina M. Jones C. Giezek H.. (2011). Long-term tolerability of telcagepant for acute treatment of migraine in a randomized trial. Headache 51, 73–84. 10.1111/j.1526-4610.2010.01799.x 21070230

[B25] Connor K. M. Shapiro R. E. Diener H. C. Lucas S. Kost J. Fan X.. (2009). Randomized, controlled trial of telcagepant for the acute treatment of migraine. Neurology 73, 970–977. 10.1212/WNL.0b013e3181b87942 19770473 PMC2754336

[B26] Crivellato E. Beltrami C. Mallardi F. Ribatti D. (2003). Paul Ehrlich’s doctoral thesis: a milestone in the study of mast cells. Br. J. Haematol. 2123, 19–21. 10.1046/j.1365-2141.2003.04573.x 14510938

[B27] Daniel B. T. (2010). Migraine. Bloomington Indiana: AuthorHouse Publishers, 101–109.

[B28] Diener H. C. Barbanti P. Dahlöf C. Reuter U. Habeck J. Podhorna J. (2011). BI 44370 TA, an oral CGRP antagonist for the treatment of acute migraine attacks: results from a phase II study. Cephalalgia 31, 573–584. 10.1177/0333102410388435 21172952

[B29] Dodick D. W. Ashina M. Brandes J. L. Kudrow D. Lanteri-Minet M. Osipova V.. (2018). ARISE: a phase 3 randomized trial of erenumab for episodic migraine. Cephalalgia 38, 1026–1037. 10.1177/0333102418759786 29471679

[B30] Dogrukol-Ak D. Tore F. Tuncel N. (2004). Passage of VIP/PACAP/secretin family across the blood-brain barrier: therapeutic effects. Curr. Pharm. Des. 10, 1325–1340. 10.2174/1381612043384934 15134484

[B31] Dong H. Zhang X. Qian Y. (2014). Mast cells and neuroinflammation. Med. Sci. Monit. Basic Res. 20, 200–206. 10.12659/MSMBR.893093 25529562 PMC4282993

[B32] Dux M. Rosta J. Sántha P. Jancsó G. (2009). Involvement of capsaicin-sensitive afferent nerves in the proteinase-activated receptor 2-mediated vasodilatation in the rat dura mater. Neuroscience 161, 887–894. 10.1016/j.neuroscience.2009.04.010 19362118

[B33] Earl N. L. McDonald S. A. Lowy M. T. (1999). The 4991W93 Investigator Group. Efficacy and tolerability of the neurogenic inflammation inhibitor, 4991W93, in the acute treatment of migraine. Cephalalgia 19:357.

[B34] Eftekhari S. Salvatore C. A. Johansson S. Chen T. B. Zeng Z. Edvinsson L. (2015). Localization of CGRP, CGRP receptor, PACAP and glutamate in trigeminal ganglion. Relation to the blood-brain barrier. Brain Res. 1600, 93–109. 10.1016/j.brainres.2014.11.031 25463029

[B35] Eller-Smith O. C. Nicol A. L. Christianson J. A. (2018). Potential mechanisms underlying centralized pain and emerging therapeutic interventions. Front. Cell. Neurosci. 12:35. 10.3389/fncel.2018.00035 29487504 PMC5816755

[B36] Erdener S. E. Dalkara T. (2014). Modelling headache and migraine and its pharmacological manipulation. Br. J. Pharmacol. 171, 4575–4594. 10.1111/bph.12651 24611635 PMC4209933

[B37] Fabbretti E. D’Arco M. Fabbro A. Simonetti M. Nistri A. Giniatullin R. (2006). Delayed upregulation of ATP P2X3 receptors of trigeminal sensory neurons by calcitonin gene-related peptide. J. Neurosci. 26, 6163–6171. 10.1523/JNEUROSCI.0647-06.2006 16763024 PMC6675180

[B38] Ferrari M. D. Odink J. Tapparelli C. Van Kempen G. M. Pennings E. J. Bruyn G. W. (1989). Serotonin metabolism in migraine. Neurology 39, 1239–1242. 10.1212/wnl.39.9.1239 2475821

[B40] Galli S. J. Tsai M. (2008). Mast cells: versatile regulators of inflammation, tissue remodeling, host defense and homeostasis. J. Dermatol. Sci. 49, 7–19. 10.1016/j.jdermsci.2007.09.009 18024086 PMC2788430

[B41] Geppetti P. Rossi E. Chiarugi A. Benemei S. (2012). Antidromic vasodilatation and the migraine mechanism. J. Headache Pain 13, 103–111. 10.1007/s10194-011-0408-3 22200764 PMC3274576

[B42] Goadsby P. J. (2013). Autonomic nervous system control of the cerebral circulation. Handb. Clin. Neurol. 117, 193–201. 10.1016/b978-0-444-53491-0.00016-x 24095126

[B43] Goadsby P. J. Edvinsson L. Ekman R. (1988). Release of vasoactive peptides in the extracerebral circulation of man and the cat during activation of the trigeminovascular system. Ann. Neurol. 23, 193–196. 10.1002/ana.410230214 2454066

[B44] Goadsby P. J. Holland P. R. Martins-Oliveira M. Hoffmann J. Schankin C. Akerman S. (2017a). Pathophysiology of migraine: a disorder of sensory processing. Physiol. Rev. 97, 553–622. 10.1152/physrev.00034.2015 28179394 PMC5539409

[B45] Goadsby P. J. Reuter U. Hallström Y. Broessner G. Bonner J. H. Zhang F.. (2017b). A controlled trial of erenumab for episodic migraine. N. Engl. J. Med. 377, 2123–2132. 10.1056/NEJMoa1705848 29171821

[B46] Gomez-Mantilla B. Cutler N. R. Leibowitz M. T. Spierings E. L. Klapper J. A. Diamond S.. (2001). Safety and efficacy of PNU-142633, a selective 5-HT1D agonist, in patients with acute migraine. Cephalalgia 21, 727–732. 10.1046/j.1468-2982.2001.00208.x 11595000

[B47] Graif Y. Shohat T. Machluf Y. Farkash R. Chaiter Y. (2018). Association between asthma and migraine: a cross-sectional study of over 110 000 adolescents. Clin. Respir. J. 12, 2491–2496. 10.1111/crj.12939 30004178

[B48] Graziottin A. Skaper S. D. Fusco M. (2014). Mast cells in chronic inflammation, pelvic pain and depression in women. Gynecol. Endocrinol. 30, 472–477. 10.3109/09513590.2014.911280 24811097

[B49] Gri G. Frossi B. D’Inca F. Danelli L. Betto E. Mion F.. (2012). Mast cell: an emerging partner in immune interaction. Front. Immunol. 3:120. 10.3389/fimmu.2012.00120 22654879 PMC3360165

[B50] Gupta K. Harvima I. T. (2018). Mast cell-neural interactions contribute to pain and itch. Immunol. Rev. 282, 168–187. 10.1111/imr.12622 29431216 PMC5812374

[B52] Headache Classification Subcommittee of the International Headache Society [IHS] (2004). The international classification of headache disorders: 2nd edition. Cephalalgia 24, 9–160. 10.1111/j.1468-2982.2003.00824.x 14979299

[B53] Heatley R. V. Denburg J. A. Bayer N. Bienenstock J. (1982). Increased plasma histamine levels in migraine patients. Clin. Allergy 12, 145–149. 10.1111/j.1365-2222.1982.tb01633.x 6176358

[B54] Hewitt D. J. Aurora S. K. Dodick D. W. Goadsby P. J. Ge Y. J. Bachman R.. (2011). Randomized controlled trial of the CGRP receptor antagonist MK-3207 in the acute treatment of migraine. Cephalalgia 31, 712–722. 10.1177/0333102411398399 21383045

[B55] Hildebrand K. A. Zhang M. Salo P. T. Hart D. A. (2008). Joint capsule mast cells and neuropeptides are increased within four weeks of injury and remain elevated in chronic stages of posttraumatic contractures. J. Orthop. Res. 26, 1313–1319. 10.1002/jor.20652 18404724 PMC2950861

[B56] Ho T. W. Ferrari M. D. Dodick D. W. Galet V. Kost J. Fan X.. (2008). Efficacy and tolerability of MK-0974 (telcagepant), a new oral antagonist of calcitonin gene-related peptide receptor, compared with zolmitriptan for acute migraine: a randomised, placebo-controlled, parallel-treatment trial. Lancet 372, 2115–2123. 10.1016/S0140-6736(08)61626-8 19036425

[B57] Idzko M. Ferrari D. Eltzschig H. K. (2014). Nucleotide signalling during inflammation. Nature 509, 310–317. 10.1038/nature13085 24828189 PMC4222675

[B58] Karatas H. Erdener S. E. Gursoy-Ozdemir Y. Lule S. Eren-Koçak E. Sen Z. D.. (2013). Spreading depression triggers headache by activating neuronal Panx1 channels. Science 339, 1092–1095. 10.1126/science.1231897 23449592

[B59] Kelleher J. H. Tewari D. McMahon S. B. (2017). Neurotrophic factors and their inhibitors in chronic pain treatment. Neurobiol. Dis. 97, 127–138. 10.1016/j.nbd.2016.03.025 27063668

[B60] Kempuraj D. Papadopoulou N. G. Lytinas M. Huang M. Kandere-Grzybowska K. Madhappan B.. (2004). Corticotropin-releasing hormone and its structurally related urocortin are synthesized and secreted by human mast cells. Endocrinology 145, 43–48. 10.1210/en.2003-0805 14576187

[B61] Khan S. Olesen A. Ashina M. (2019). CGRP, a target for preventive therapy in migraine and cluster headache: systematic review of clinical data. Cephalalgia 39, 374–389. 10.1177/0333102417741297 29110503

[B62] Kilinc E. Dagistan Y. Kukner A. Yilmaz B. Agus S. Soyler G.. (2018). Salmon calcitonin ameliorates migraine pain through modulation of CGRP release and dural mast cell degranulation in rats. Clin. Exp. Pharmacol. Physiol. 45, 536–546. 10.1111/1440-1681.12915 29344989

[B63] Kilinc E. Firat T. Tore F. Kiyan A. Kukner A. Tunçel N. (2015). Vasoactive Intestinal peptide modulates c-Fos activity in the trigeminal nucleus and dura mater mast cells in sympathectomized rats. J. Neurosci. Res. 93, 644–650. 10.1002/jnr.23523 25476208

[B64] Kilinc E. Guerrero-Toro C. Zakharov A. Vitale C. Gubert-Olive M. Koroleva K.. (2017). Serotonergic mechanisms of trigeminal meningeal nociception: implications for migraine pain. Neuropharmacology 116, 160–173. 10.1016/j.neuropharm.2016.12.024 28025094

[B65] Klede M. Clough G. Lischetzki G. Schmelz M. (2003). The effect of the nitric oxide synthase inhibitor N-nitro-L-arginine-methyl ester on neuropeptide-induced vasodilation and protein extravasation in human skin. J. Vasc. Res. 40, 105–114. 10.1159/000070707 12808346

[B67] Körtési T. Tuka B. Tajti J. Bagoly T. Fülöp F. Helyes Z.. (2018). Kynurenic acid inhibits the electrical stimulation induced elevated pituitary adenylate cyclase-activating polypeptide expression in the TNC. Front. Neurol. 8:745. 10.3389/fneur.2017.00745 29387039 PMC5775965

[B68] Kunkle E. C. (1959). Acetylcholine in the mechanism of headaches of migraine type. AMA Arch. Neurol. Psychiatry 81, 135–141. 10.1001/archneurpsyc.1959.02340140001001 13616769

[B69] Lassen L. H. Thomsen L. L. Olesen J. (1995). Histamine induces migraine via the H1-receptor. Support for the NO hypothesis of migraine. Neuroreport 6, 1475–1479. 10.1097/00001756-199507310-00003 7579128

[B70] Lennerz J. K. Rühle V. Ceppa E. P. Neuhuber W. L. Bunnett N. W. Grady E. F.. (2008). Calcitonin receptor-like receptor (CLR), receptor activity-modifying protein 1 (RAMP1), and calcitonin gene-related peptide (CGRP) immunoreactivity in the rat trigeminovascular system: differences between peripheral and central CGRP receptor distribution. J. Comp. Neurol. 507, 1277–1299. 10.1002/cne.21607 18186028

[B71] Levy D. (2012). Endogenous mechanisms underlying the activation and sensitization of meningeal nociceptors: the role of immuno-vascular interactions and cortical spreading depression. Curr. Pain Headache Rep. 16, 270–277. 10.1007/s11916-012-0255-1 22328144

[B72] Levy D. Burstein R. Kainz V. Jakubowski M. Strassman A. M. (2007). Mast cell degranulation activates a pain pathway underlying migraine headache. Pain 130, 166–176. 10.1016/j.pain.2007.03.012 17459586 PMC2045157

[B73] Levy D. Burstein R. Strassman A. M. (2005). Calcitonin gene-related peptide does not excite or sensitize meningeal nociceptors: implications for the pathophysiology of migraine. Ann. Neurol. 58, 698–705. 10.1002/ana.20619 16240341

[B74] Levy D. Labastida-Ramirez A. MaassenVanDenBrink A. (2018). Current understanding of meningeal and cerebral vascular function underlying migraine headache. Cephalalgia [Epub ahead of print]. 10.1177/0333102418771350 29929378

[B75] Luger T. A. Lotti T. (1998). Neuropeptides: role in inflammatory skin diseases. J. Eur. Acad. Dermatol. Venereol. 10, 207–211. 10.1016/s0926-9959(98)00009-9 9643321

[B76] Malone C. D. Bhowmick A. Wachholtz A. B. (2015). Migraine: treatments, comorbidities, and quality of life, in the USA. J. Pain Res. 8, 537–547. 10.2147/jpr.s88207 26316804 PMC4540217

[B77] Marcus R. Goadsby P. J. Dodick D. Stock D. Manos G. Fischer T. Z. (2014). BMS-927711 for the acute treatment of migraine: a double-blind, randomized, placebo controlled, dose-ranging trial. Cephalalgia 34, 114–125. 10.1177/0333102413500727 23965396

[B78] Messlinger K. (2018). The big CGRP flood—sources, sinks and signalling sites in the trigeminovascular system. J. Headache Pain 19:22. 10.1186/s10194-018-0848-0 29532195 PMC5847494

[B79] Metcalfe D. D. Baram D. Mekori Y. A. (1997). Mast cells. Physiol. Rev. 77, 1033–1079. 10.1152/physrev.1997.77.4.1033 9354811

[B80] Mikhailov N. Mamontov O. Kamshilin A. Giniatullin R. (2016). Parasympathetic cholinergic and neuropeptide mechanisms of migraine. Anesth. Pain Med. 7:e42210. 10.5812/aapm.42210 28920040 PMC5554415

[B81] Nilsson G. Costa J. J. Metcalfe D. D. Gallin J. I. Snyderman R. (1999). “Mast cells and basophils,” in Inflammation: Basic Principles and Clinical Correlates, eds Gallin J. I. Snyderman R. (Philadelphia: Lippincott-Raven), 97–117.

[B51] Nurkhametova D. Kudryavtsev I. Giniatullina V. Serebryakova M. Giniatullina R. R. Wojciechowski S.. (2019). Extracellular ATP induces activation and degranulation of meningeal mast cells through P2X7 receptor: a possible mechanism for migraine pain. Front. Cell. Neurosci. 13:45. 10.3389/fncel.2019.00045 30814932 PMC6381076

[B82] Okragly A. J. Morin S. M. DeRosa D. Martin A. P. Johnson K. W. Johnson M. P.. (2018). Human mast cells release the migraine-inducing factor pituitary adenylate cyclase-activating polypeptide (PACAP). Cephalalgia 38, 1564–1574. 10.1177/0333102417740563 29103295

[B83] Olesen J. (2008). The role of nitric oxide (NO) in migraine, tension-type headache and cluster headache. Pharmacol. Ther. 120, 157–171. 10.1016/j.pharmthera.2008.08.003 18789357

[B84] Olesen J. Diener H. C. Husstedt I. W. Goadsby P. J. Hall D. Meier U.. (2004). Calcitonin gene-related peptide receptor antagonist BIBN 4096 BS for the acute treatment of migraine. N. Engl. J. Med. 350, 1104–1110. 10.1056/NEJMoa030505 15014183

[B85] Olness K. Hall H. Rozniecki J. J. Schmidt W. Theoharides T. C. (1999). Mast cell activation in children with migraine before and after training in self-regulation. Headache 39, 101–107. 10.1046/j.1526-4610.1999.3902101.x 15613202

[B86] Peroutka S. J. (2004). Migraine: a symphatetic nervous system disorder. Headache 44, 53–64. 10.1111/j.1526-4610.2004.04011.x 14979884

[B87] Peroutka S. J. (2005). Neurogenic inflammation and migraine: implications for the therapeutics. Mol. Interv. 5, 304–311. 10.1124/mi.5.5.10 16249526

[B88] Pietrobon D. Striessnig J. (2003). Neurobiology of migraine. Nat. Rev. Neurosci. 4, 386–398. 10.1038/nrn1102 12728266

[B89] Pietrobon D. Moskowitz M. A. (2013). Pathophysiology of migraine. Annu. Rev. Physiol 75, 365–391. 10.1146/annurev-physiol-030212-183717 23190076

[B90] Ramachandran R. Bhatt D. K. Ploug K. B. Hay-Schmidt A. Jansen-Olesen I. Gupta S.. (2014). Nitric oxide synthase, calcitonin gene-related peptide and NK-1 receptor mechanisms are involved in GTN-induced neuronal activation. Cephalalgia 34, 136–147. 10.1177/0333102413502735 24000375

[B91] Recober A. Russo A. F. (2009). Calcitonin gene-related peptide: an update on the biology. Curr. Opin. Neurol. 22, 241–246. 10.1097/WCO.0b013e32832b2427 19434786 PMC2844706

[B92] Reuter U. Goadsby P. J. Lanteri-Minet M. Wen S. Hours-Zesiger P. Ferrari M. D.. (2018). Efficacy and tolerability of erenumab in patients with episodic migraine in whom two-to-four previous preventive treatments were unsuccessful: a randomised, double-blind, placebo-controlled, phase 3b study. Lancet 392, 2280–2287. 10.1016/s0140-6736(18)32534-0 30360965

[B93] Rosa A. C. Fantozzi R. (2013). The role of histamine in neurogenic inflammation. Br. J. Pharmacol. 170, 38–45. 10.1111/bph.12266 23734637 PMC3764847

[B94] Rossi P. Fiermonte G. Pierelli F. (2003). Cinnarizine in migraine prophylaxis: efficacy, tolerability and predictive factors for therapeutic responsiveness. An open-label pilot trial. Funct. Neurol. 18, 155–159. 10.1007/s10194-008-0013-2 14703897

[B95] Rozniecki J. J. Dimitriadou V. Lambracht-Hall M. Pang X. Theoharides T. C. (1999). Morphological and functional demonstration of rat dura mater mast cell-neuron interactions *in vitro* and *in vivo*. Brain Res. 849, 1–15. 10.1016/s0006-8993(99)01855-7 10592282

[B96] Russo A. F. Dickerson I. M. (2006). “CGRP: a multifunctional neuropeptide,” in Handboook Neurochem Molec Neurobiol, (Vol. 3) ed. Lajtha A. (New York, NY: Springer), 391–426.

[B98] Schwenger N. Dux M. de Col R. Carr R. Messlinger K. (2007). Interaction of calcitonin gene-related peptide, nitric oxide and histamine release in neurogenic blood flow and afferent activation in the rat cranial dura mater. Cephalalgia 27, 481–491. 10.1111/j.1468-2982.2007.01321.x 17441973

[B99] Schytz H. W. (2010). Investigation of carbachol and PACAP38 in a human model of migraine. Dan. Med. Bull. 57:B4223. 10.1007/BF02529865 21122466

[B100] Shelukhina I. Mikhailov N. Abushik P. Nurullin L. Nikolsky E. E. Giniatullin R. (2017). Cholinergic nociceptive mechanisms in rat meninges and trigeminal ganglia: potential implications for migraine pain. Front. Neurol. Shelukhina:163. 10.3389/fneur.2017.00163 28496430 PMC5406407

[B101] Shepheard S. Edvinsson L. Cumberbatch M. Williamson D. Mason G. Webb J.. (1999). Possible antimigraine mechanisms of action of the 5HT1F receptor agonist LY334370. Cephalalgia 19, 851–858. 10.1046/j.1468-2982.1999.1910851.x 10668103

[B102] Shevel E. (2011). The extracranial vascular theory of migraine–a great story confirmed by the facts. Headache 51, 409–417. 10.1111/j.1526-4610.2011.01844.x 21352215

[B103] Sicuteri F. (1963). Mast cells and their active substances: their role in the pathogenesis of migraine. Headache 3, 86–92. 10.1111/j.1526-4610.1963.hed0303086.x 14094109

[B104] Sismanopoulos N. Delivanis D. A. Alysandratos K. D. Angelidou A. Therianou A. Kalogeromitros D.. (2012). Mast cells in allergic and inflammatory diseases. Curr. Pharm. Des. 18, 2261–2277. 10.2174/138161212800165997 22390690

[B105] Skaper S. D. (2017). Nerve growth factor: a neuroimmune crosstalk mediator for all seasons. Immunology 151, 1–15. 10.1111/imm.12717 28112808 PMC5382350

[B106] Tepper S. J. (2018). History and review of anti-calcitonin gene-related peptide (CGRP) therapies: from translational research to treatment. Headache 58, 238–275. 10.1111/head.13379 30242830

[B109] Theoharides T. C. Alysandratos K. D. Angelidou A. Delivanis D. A. Sismanopoulos N. Zhang B.. (2012). Mast cells and inflammation. Biochim. Biophys. Acta 1822, 21–33. 10.1016/j.bbadis.2010.12.014 21185371 PMC3318920

[B108] Theoharides T. C. Donelan J. Kandere-Grzybowska K. Konstantinidou A. (2005). The role of mast cells in migraine pathophysiology. Brain Res. Rev. 49, 65–76. 10.1016/j.brainresrev.2004.11.006 15960987

[B107] Theoharides T. C. Kalogeromitros D. (2006). The critical role of mast cells in allergy and inflammation. Ann. N Y Acad. Sci. 1088, 78–99. 10.1196/annals.1366.025 17192558

[B113] Tuka B. Helyes Z. Markovics A. Bagoly T. Nemeth J. Mark L.. (2012). Peripheral and central alterations of pituitary adenylate cyclase activating polypeptide-likeimmunoreactivity in the rat in response to activation of the trigeminovascular system. Peptides 33, 307–316. 10.1016/j.peptides.2011.12.019 22245521

[B114] Tuka B. Helyes Z. Markovics A. Bagoly T. Szolcsányi J. Szabó N.. (2013). Alterations in PACAP-38-like immunoreactivity in the plasma during ictal and interictal periods of migraine patients. Cephalalgia 33, 1085–1095. 10.1177/0333102413483931 23598374

[B110] Tore F. Korkmaz O. T. Dogrukol-Ak D. Tunçel N. (2010). The effects of vasoactive ıntestinal peptide on dura mater nitric oxide levels and vessel-contraction responses in sympathectomized rats. J. Mol. Neurosci. 41, 288–293. 10.1007/s12031-009-9310-8 19936638

[B111] Tore F. Tuncel N. (2009). Mast cells: target and source of neuropeptides. Curr. Pharm. Des. 15, 3433–3445. 10.2174/138161209789105036 19860689

[B112] Tore F. Tuncel N. (2011). Anatomical and functional relationships between sensory nerves and mast cells. AIAAA Med. Chem. 10, 10–17. 10.2174/187152311795325550

[B115] van Dongen R. M. Zielman R. Noga M. Dekkers O. M. Hankemeier T. van den Maagdenberg A. M.. (2017). Migraine biomarkers in cerebrospinal fluid: a systematic review and meta-analysis meta-analysis. Cephalalgia 37, 49–63. 10.1177/0333102415625614 26888294

[B116] Varatharaj A. Mack J. Davidson J. R. Gutnikov A. Squier W. (2012). Mast cells in the human dura: effects of age and dural bleeding. Childs Nerv. Syst. 28, 541–545. 10.1007/s00381-012-1699-7 22270653

[B117] Vincent L. Vang D. Nguyen J. Gupta M. Luk K. Ericson M. E.. (2013). Mast cell activation contributes to sickle cell pathobiology and pain in mice. Blood 122, 1853–1862. 10.1182/blood-2013-04-498105 23775718 PMC3772495

[B118] Voss T. Lipton R. B. Dodick D. W. Dupre N. Ge J. Y. Bachman R.. (2016). A phase IIb randomized, double-blind, placebo-controlled trial of ubrogepant for the acute treatment of migraine. Cephalalgia 36, 887–898. 10.1177/0333102416653233 27269043

[B119] Waeber C. Moskowitz M. A. (2005). Migraine as an inflammatory disorder. Neurology 64, S9–S15. 10.1212/WNL.64.10_suppl_2.S9 15911785

[B120] Wareham K. J. Seward E. P. (2016). P2X7 receptors induce degranulation in human mast cells. Purinergic Signal. 12, 235–246. 10.1007/s11302-016-9497-4 26910735 PMC4854833

[B121] Watson J. J. Allen S. J. Dawbarn D. (2008). Targeting nerve growth factor in pain: what is the therapeutic potential? BioDrugs 22, 349–359. 10.2165/0063030-200822060-00002 18998753

[B122] Wöber-Bingöl C. (2013). Epidemiology of migraine and headache in children and adolescents. Curr. Pain Headache Rep. 17:341. 10.1007/s11916-013-0341-z 23700075

[B123] Xu Y. Chen G. (2015). Mast cell and autoimmune diseases. Mediators Inflamm. 2015:246126. 10.1155/2015/246126 25944979 PMC4402170

[B124] Yan L. Dong X. Xue L. Xu H. Zhou Z. Wan Q. (2018). Neurogenic dural inflammation induced by inflammatory soup combined with CGRP: a modified animal model of migraine. Int. J. Clin. Exp. Med. 11, 9126–9134.

[B125] Yarnitsky D. Goor-Aryeh I. Bajwa Z. H. Ransil B. I. Cutrer F. M. Sottile A.. (2003). Wolff Award: possible parasympathetic contributions to peripheral and central sensitization during migraine. Headache 43, 704–714. 10.1046/j.1526-4610.2003.03127.x 12890124

[B126] Yegutkin G. G. Guerrero-Toro C. Kilinc E. Koroleva K. Ishchenko Y. Abushik P.. (2016). Nucleotide homeostasis and purinergic nociceptive signaling in rat meninges in migraine-like conditions. Purinergic Signal. 12, 561–574. 10.1007/s11302-016-9521-8 27369815 PMC5023636

[B127] Yildiz S. K. Turkoglu S. A. Yildiz N. Ozturk A. Tore F. (2007). Sympathetic skin responses of the face and neck evoked by electrical stimulation. Auton. Neurosci. 134, 85–91. 10.1016/j.autneu.2007.02.005 17383240

[B128] Yildiz S. K. Yildiz N. Korkmaz B. Altunrende B. Gezici A. R. Alkoy S. (2008). Sympathetic skin responses from frontal region in migraine headache: a pilot study. Cephalalgia 28, 696–704. 10.1111/j.1468-2982.2008.01574.x 18460010

[B129] Zakharov A. Vitale C. Kilinc E. Koroleva K. Fayuk D. Shelukhina I.. (2015). Hunting for origins of migraine pain: cluster analysis of spontaneous and capsaicin-induced firing in meningeal trigeminal nerve fibers. Front. Cell. Neurosci. 9:287. 10.3389/fncel.2015.00287 26283923 PMC4516892

[B131] Zhang X. C. Levy D. (2008). Modulation of meningeal nociceptors mechanosensitivity by peripheral proteinase-activated receptor-2: the role of mast cells. Cephalalgia 28, 276–284. 10.1111/j.1468-2982.2007.01523.x 18254896 PMC2504502

[B130] Zhang X. Strassman A. M. Burstein R. Levy D. (2007). Sensitization and activation of intracranial meningeal nociceptors by mast cell mediators. J. Pharmacol. Exp. Ther. 322, 806–812. 10.1124/jpet.107.123745 17483291

